# PNO1 regulates autophagy and apoptosis of hepatocellular carcinoma via the MAPK signaling pathway

**DOI:** 10.1038/s41419-021-03837-y

**Published:** 2021-05-28

**Authors:** Zhiqiang Han, Dongming Liu, Lu Chen, Yuchao He, Xiangdong Tian, Lisha Qi, Liwei Chen, Yi Luo, Ziye Chen, Xiaomeng Hu, Guangtao Li, Linlin Zhan, Yu Wang, Qiang Li, Peng Chen, Zhiyong Liu, Hua Guo

**Affiliations:** 1 Department of Tumor Cell Biology, Tianjin Medical University Cancer Institute and Hospital, 300060 Tianjin, China; 2 Department of Hepatobiliary Cancer, Liver Cancer Research Center, Tianjin Medical University Cancer Institute and Hospital, 300060 Tianjin, China; 3 Tianjin Medical University Cancer Institute and Hospital, National Clinical Research Center for Cancer, Key Laboratory of Cancer Prevention and Therapy, Tianjin’s Clinical Research Center for Cancer, 300060 Tianjin, China; 4 Department of Endoscopy, Tianjin Medical University Cancer Institute and Hospital, 300060 Tianjin, China; 5 Department of Pathology, Tianjin Medical University Cancer Institute and Hospital, 300060 Tianjin, China; 6 Department of Thoracic Oncology, Lung Cancer Diagnosis and Treatment Center, Tianjin Medical University Cancer Institute and Hospital, 300060 Tianjin, China

**Keywords:** Cancer genomics, Cancer genomics

## Abstract

Some studies have reported that activated ribosomes are positively associated with malignant tumors, especially in hepatocellular carcinoma (HCC). The RNA-binding protein PNO1 is a critical ribosome rarely reported in human tumors. This study aimed to explore the molecular mechanisms of PNO1 in HCC. Using 150 formalin-fixed and paraffin-embedded samples and 8 fresh samples, we found high PNO1 expression in HCC tumor tissues through Western blotting and RT-PCR. Moreover, the higher PNO1 expression was associated with poor HCC prognosis patients. In vitro and in vivo experiments indicated that PNO1 overexpression promoted the proliferation and depressed the apoptosis of HCC cells. High PNO1 expression also increased the autophagy of HCC cells. The molecular mechanisms underlying PNO1 were examined by RNA-seq analysis and a series of functional experiments. Results showed that PNO1 promoted HCC progression through the MAPK signaling pathway. Therefore, PNO1 was overexpressed in HCC, promoted autophagy, and inhibited the apoptosis of HCC cells through the MAPK signaling pathway.

## Introduction

Hepatocellular carcinoma (HCC) is the fourth most common cause of cancer-related mortality worldwide^1^. More than 380,000 people die of liver cancer in China each year, accounting for 51% of the global HCC-affected population^2^. The high recurrence and metastasis rate explain the poor prognosis of HCC patients, even though the treatment of HCC is constantly updated and improved^3,4^. Therefore, novel molecules should be explored to improve HCC prognosis.

Targeting apoptosis represents a potent cancer-treatment strategy^5,6^. Apoptosis, one of the cancer hallmarks, is a biological process of energy dependence, cellular autonomy, and orderliness^7,8^. Caspases are ubiquitously expressed cysteine proteases that play a central role in apoptosis^9^. Death-inducing stimuli lead to cleavage at the aspartic residues of caspases and removal of the N-terminal inhibitory domain, resulting in the demolition phase of apoptosis^10,11^. Apoptosis is the main and most well-studied form of programmed cell death. However, autophagy is a distinct mode of cell death associated with the generation of energy and metabolites through the digestion of intracellular macromolecules and organelles. Several studies have indicated that autophagy plays a dual role in cancer^12^. On one hand, autophagy serves as a suppressor in the early stage of cancer development by inhibiting inflammation and promoting genomic stability. On the other hand, autophagy can promote tumorigenesis and angiogenesis by supplying nutrients and energy^13,14^.

Our previous study has shown that the RNA-binding protein “partner of NOB1” (PNO1) promotes the proliferation, invasion, and metastasis of lung adenocarcinoma through the miR-340-5p/Notch biological axis^15^. PNO1 gene is located in human chromosome 2p14 and comprises seven exons and six introns, thereby playing an essential role in ribosome biogenesis and promoting the maturation of small ribosomal subunits^16–18^. Many studies have shown that imbalance in the ribosome-biogenesis process is related to tumor progression^19–22^. Some studies have demonstrated the relationship of apoptosis and autophagy with ribosome biogenesis^23–25^, but the specific mechanism in tumors remains unclear.

In addition to the function of PNO1 in ribosome biogenesis, recent studies have reported that PNO1 may affect the progression of colorectal cancer (CRC) and urinary bladder carcinoma^26–28^. However, the role of PNO1 in tumor progression and its specific regulatory mechanism remain unclear. In the current study, we demonstrated that PNO1 was overexpressed in HCC tissues and may act as a specific prognostic biomarker of HCC. Furthermore, we demonstrated that PNO1 can inhibit the apoptosis of HCC cells by promoting autophagy through the Erk/MAPK signaling pathway in vivo and in vitro. Our results unraveled a novel function of PNO1 in tumor cells and can thus serve as a new therapeutic strategy for targeting PNO1 in HCC.

## Materials and methods

### Western blot and antibodies

The cells were lysed with 1 × SDS lysis buffer (Tris–HCl, pH 6.8, 62.5 mM, 2% SDS, 10% glycerol) supplemented with 1 mM NaF, 1 mM Na_3_VO_4_, 1 × protease, and phosphatase inhibitor cocktail (Hoffman-la Roche Ltd, Basel, Switzerland) on ice for 30 min. The collected protein was denatured in a 95 °C water bath for 10 min and centrifuged at 12,000 rpm at 4 °C for 10 min, then the upper clear cell lysates were transferred to new tubes. Equal amounts of protein were loaded on gels and separated by SDS–PAGE. Then, proteins were transferred to PVDF membranes (Immobilon-P; Millipore, Billerica, Massachusetts) and blocked with 5% milk or bovine serum albumin, followed by incubation with primary and secondary antibodies. The following antibodies were used: anti-PNO1 (1:1000) from Santa Cruz Biotechnology, anti-β-actin (1:1000),anti-Atg7 (1:1000), anti-Atg5 (1:1000), anti-LC3B (1:1000), anti-SQSTM1/p62 (1:1000), anti-Beclin 1 (1:1000), anti-Erk (1:1000), anti-p-Erk (1:1000), anti-p38 (1:1000), anti-p-p38 (1:1000) from Cell Signaling Technology (Beverly, MA), anti-caspase 3 (1:500), anti-caspase 9 (1:300) from Proteintech (Chicago, IL, USA), and anti-Bcl-2 (1:500), anti-Bax (1:1000) from Bioss (Beijing, China).

### Patients and tissue specimens

A total of 150 patients with HCC surgically resected between January 2010 and December 2014 and with available formalin-fixed paraffin-embedded (FFPE) tumor samples were retrospectively identified at Tianjin Medical University Cancer Institute and Hospital (Tianjin, China). This study was consistent with the ethical guidelines of the Helsinki Declaration and approved by the Ethics Committee.

### Cell culture

Hep3B and HLE cells were purchased from American Type Culture Collection (ATCC, Manassas, VI). Cells were cultivated in 1640 medium (Corning, NY, USA), supplemented with 1% penicillin/streptomycin (PS; HyClone, Logan, UT, USA) and 10% fetal bovine serum (FBS; PAN-Seratech, Edenbach, Germany), and the incubating temperature was 37 °C, with 5% CO_2_.

### Cell transfection

To obtain lentiviral particles, packaging plasmids (VSVG and ΔR) and expression plasmids (sh-PNO1, sh-Ctrl, PNO1, and Vector) were transfected into HEK293T cells using Lipofectamine 2000 (Invitrogen). The lentiviruses were produced by HEK293T cells. Hep3B and HLE cells were infected with a lentivirus to produce stable PNO1 KD or OE cells.

### Transcriptome sequencing analysis

Transcriptome sequencing was based on the Illumina sequencing platform to analyze gene expression of Hep3B sh-PNO1 cells and sh-Ctrl cells. DEGs were filtered by log2 (fold change) ≥ 0 and *p*-value ≤ 0.05. ClusterProfiler was used for gene enrichment analysis of gene ontology and KEGG, and *p*-value ≤ 0.05 was used as the enrichment cutoff.

### Immunohistochemistry

The human HCC tissue microarrays and xenograft tumor tissue sections were deparaffinized in xylene, rehydrated through an ethanol series, and antigen was then retrieved in citrate. Treated with 3% hydrogen peroxide to inhibit endogenous peroxide activities for 10 min. Then samples were stained using antibodies at room temperature for 30 min and overnight at 4 °C. After washing, tissue microarrays and sections were incubated with secondary antibody for 1 h at room temperature. Visualized with 3,3-diaminobenzidine solution (ZSGB-Bio) treatment and counterstained with hematoxylin. The percentage immunoreactivity score was classified on a 4-point scale: 0. <10% positive cells; 1. 10–40% positive cells; 2. 40–70% positive cells; and 3. 70–100% positive cells.

### Flow cytometric analysis of cell apoptosis

Cells were digested and resuspended in 10 μl 1× binding buffer. Stained with 5 μl Annexin V and 5 μl PI (eBioscience, CA, USA) for 15 min at room temperature (25 °C) in the dark. The number of apoptotic cells was analyzed by using FACS Aria flow cytometer with CellQuest software and the data were analyzed with FlowJo software.

### Immunofluorescence

Cells were seeded in six-well plates and fixed in 4% PFA. For tissue immunofluorescence experiments, tumor tissue was isolated from xenograft model, fixed in 4% PFA, embedded in paraffin, and then cut into 5-μm sections for immunofluorescence staining. Sections were blocked with 10% bovine serum albumin and incubated with primary antibody. This was followed by incubation with fluorescently labeled secondary antibodies. Nuclei were labeled with 4′,6-diamidino-2-phenylindole (DAPI) (Beyotime, Shanghai), and cells were visualized using fluorescence microscopy.

### Autophagic flux counting

Cells were transfected with the stubRFP-sensGFP-LC3 lentivirus purchased from GENE-CHEM following the manufacturer’s protocol. After transfection with mRFP-GFP-LC3, autophagosomes were labeled yellow (mRFP and GFP) whereas autolysosomes were labeled red (mRFP only). Then cells were visualized using fluorescence microscopy.

### Animal models

For the animal experiments, male BALB/c nude mice (4 weeks old) were purchased from SPF Biotechnology (Beijing, China). Prepared 5 × 10^6^ tumor cells at the volume of 100 μL were injected into the subcutaneous tissue of each mice by a 1 mL injector (*n* = 9 in HLE PNO1 and Vector group, *n* = 5 in Hep3B sh-PNO1 and sh-Ctrl group). The status of the mice was observed every 2 days. The tumor volume was checked with a caliper, and the variation of weight of the mice was recorded by a scale.

### Cell viability assay

Cells were plated in a 96-well plate at 2000 cells/well. Then, added 10 μL of CCK-8 reagent (Dojindo) in each well and coincubated with the cells in a 37 °C incubator for 4 h. The OD value of the wavelength at 450 nm was measured by an enzyme labeling instrument. The cell proliferation curve was drawn by continuous detection for 3–4 days.

### Colony formation assay

For the colony formation assay, 1000 cells in DMEM supplemented with 10% FBS were plated in six-well plates. After 2 weeks of incubation, the surviving colonies were fixed, stained with 0.5% crystal violet, imaged, and counted, and the data are presented as the means ± SDs of triplicate dishes in the same experiment.

### TCGA and GEO datasets

We downloaded the raw data of TCGA (National Cancer Institute) and GEO (NCBI) related to HCC on the official website. Then the data was normalized by R Studio. We analyzed the expression of PNO1 in matched tumor and para-tumor of TCGA and GEO database (GSE54236). Further analysis of the expression level of PNO1 with the prognosis of HCC patients.

### Gene set enrichment analysis

GSEA was performed to determine whether the PNO1 mRNA level is related to biological states, including proliferation, autophagy, apoptosis, and the high expression of some genes in the tumor, on the basis of GSE54236 and GSE45436 data sets for HCC using GSEA 4.1.0 (The Broad Institute of MIT and Harvard).

### Statistical analyses

SPSS 26.0 (SPSS Inc., Chicago, IL) was used to evaluate the data. The univariate Kaplan–Meier method and multivariate Cox method were used to analyze the independent risk factors and survival curve. Statistical differences were analyzed with a two-tailed Student’s *t*-test or one-way ANOVA. Statistical significance was defined as *p* < 0.05.

## Results

### PNO1 was overexpressed in HCC tissues and associated with poor prognosis of HCC patients

**Fig. 1:**
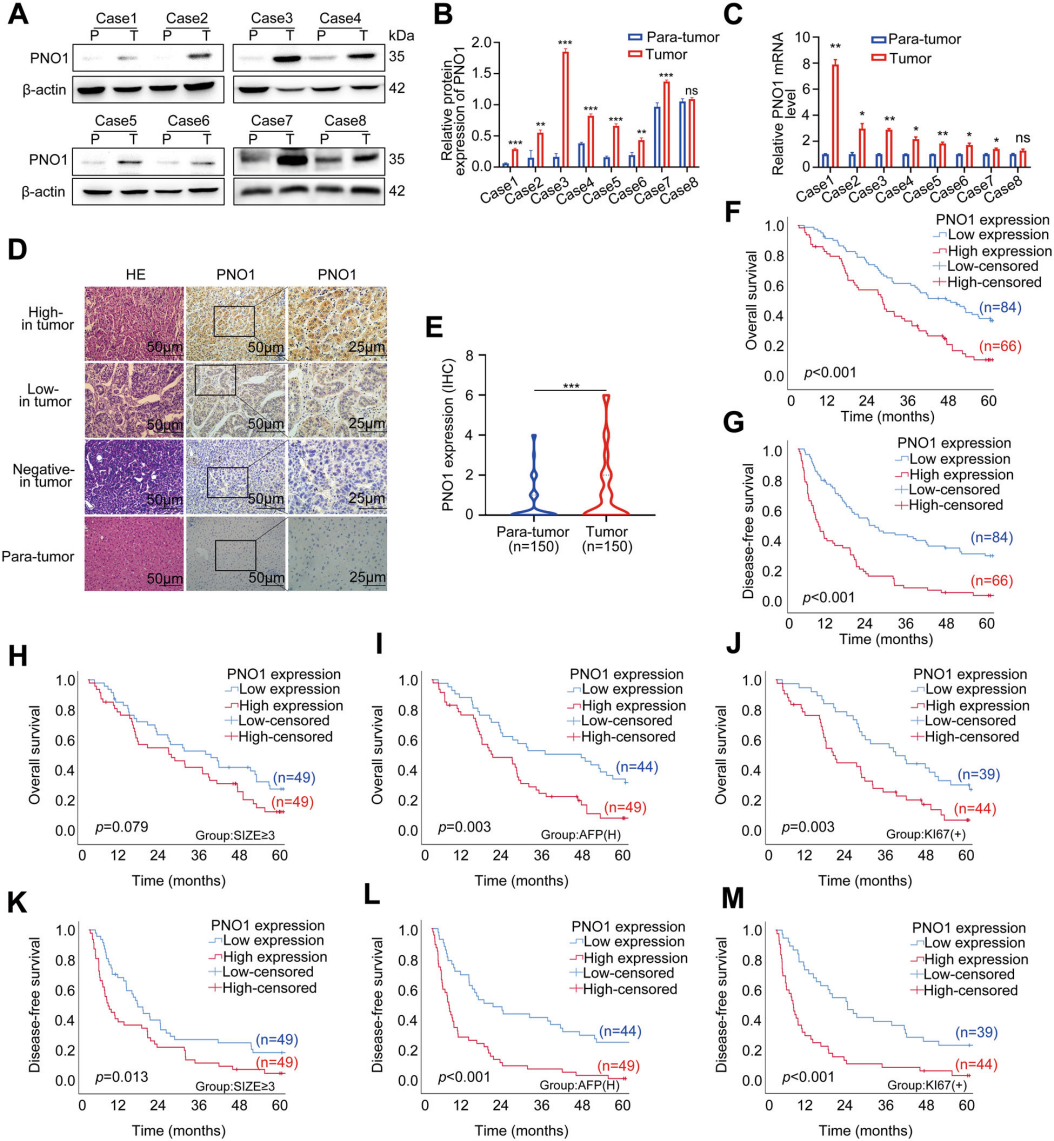
PNO1 expression is elevated in HCC tissue samples and predicts poor survival in HCC patients. **A**–**C** Western Blotting and RT-PCR showing the differences in the PNO1 protein and mRNA levels between eight pairs of HCC tissue (T) and adjacent normal tissue (P). **D**, **E** IHC analysis of PNO1 expression in 150 pairs tissue microarrays of HCC specimens. Representative images were taken at a magnification of 50 or 25 μm, *p* < 0.05. **F**, **G** The Kaplan–Meier survival analysis of overall survival (**F**) and disease-free survival (**G**) for 150 HCC patients. **H**, **K** The Kaplan–Meier survival analysis of overall survival (**H**) and disease-free survival (**K**) for HCC patients in tumor size ≥ 3 cm subgroup. **I**, **L** The Kaplan–Meier survival analysis of overall survival (**I**) and disease-free survival (**L**) for HCC patients in high serum AFP level subgroup. **J**, **M** The Kaplan–Meier survival analysis of overall survival (**J**) and disease-free survival (**M**) for HCC patients in a more positive rate of Ki-67 subgroup. Data were presented as mean ± SEM. *n* = 3–4. *p < 0.05, **p < 0.01, ***p < 0.001.

Our previous study has shown that PNO1 is highly expressed in lung adenocarcinoma tissues and is closely related to poor prognosis. To further investigate the expression pattern of PNO1 in HCC, we detected PNO1 expression in HCC tissues. The protein level of PNO1 in eight pairs of fresh HCC tissue samples was considerably higher than that in adjacent normal tissue samples (Fig. 1A and B). Moreover, RT-PCR results showed a similar tendency (Fig. 1C). To further explore the expression pattern of PNO1 in HCC patients, a large cohort of 150 HCC samples tissue microarray was evaluated by immunohistochemistry. The findings demonstrated that PNO1 was overexpressed in HCC tumor tissues (*p* < 0.05, Fig. 1D and E). Furthermore, the increased PNO1 expression was associated with poorer overall survival (OS) and disease-free survival (DFS) (*p* < 0.001, Fig. 1F and G). Patients with low expression of PNO1 had a 5-year OS rate of 40.2%, which was much better than that of 13.7% for patients with high PNO1 expression. The Cox regression model showed that increased PNO1 expression was an independent risk factor for the survival of HCC patients (Table 1). Moreover, multivariate-analysis results for HCC patients’ survival are presented in the forest plots (Supplementary Fig. 1A and B).

**Table 1:** Univariate and multivariate analysis of prognostic factors associated with OS and DFS in 150 HCC patients.

HCC patients (*n* = 150)	Univariate analysis of OS			Multivariate analysis of OS			Univariate analysis of DFS			Multivariate analysis of DFS		
	HR (log rank)	95% CI	*P*-value	HR (log rank)	95% CI	*P*-value	HR (log rank)	95% CI	*P*-value	HR (log rank)	95% CI	*P*-value
*Age (years)* ≥55/<55	0.926	0.630–1.362	0.695				1.268	0.791–2.034	0.609			
*Sex* Male/female	0.877	0.556–1.384	0.573				0.795	0.514–1.229	0.555			
*AFP (ng/ml)* ≥7/<7	1.727	1.145–2.605	0.008*	1.477	0.953–2.289	0.081	1.991	1.242–3.192	0.004*	1.303	0.868–1.958	0.202
*Staining score of KI67* ≥5%/<5%	1.732	1.164–2.578	0.006*	1.644	1.078–2.507	0.021*	1.760	1.130–2.741	0.032*	1.321	0.896–1.947	0.160
*HBV* Y/N	1.429	0.876–2.329	0.150				1.768	1.092–2.862	0.143			
*Tumor size (cm)* >3/≤3	1.545	1.018–2.345	0.039*	1.358	0.860–2.144	0.189	2.489	1.528–4.056	0.014*	1.146	0.737–1.782	0.544
*Mavi* Y/N	4.238	2.391–7.511	<0.001*	1.556	0.565–4.282	0.392	1.519	0.780–2.956	<0.001*	1.558	0.613–3.957	0.351
*Mivi* Y/N	1.634	1.105–2.418	0.013*	1.412	0.946–2.107	0.091	0.940	0.606–1.459	0.025*	1.487	1.027–2.152	0.036*
*Cirrhosis* Y/N	0.933	0.633–1.374	0.723				0.999	0.584–1.70-	0.701			
*BCLC stage* 0&A/B&C	3.036	1.865–4.940	<0.001*	2.244	0.924–5.446	0.074	0.984	0.576–1.680	<0.001*	2.487	1.099–5.626	0.029*
*Staining* *score of PNO1* High/low	2.098	1.419–3.103	<0.001*	1.703	1.111–2.609	0.014*	0.535	0.316–0.906	<0.001*	2.421	1.605–3.651	<0.001*

Bold and asterisks are used to emphasize that the value is statistically significant.

We also analyzed the correlation between PNO1 expression and other clinical indicators. However, no significant correlation existed between PNO1 expression and routine clinicopathological features, such as age, gender, HBV infection, microvascular invasion, macrovascular invasion, cirrhosis, and BCLC stage. Notably, high PNO1 expression was marginally significantly related with tumor size (*p* = 0.042), serum alpha-fetoprotein (AFP) level (*p* = 0.006), and positive rate of Ki-67 (*p* = 0.013) (Supplementary Fig. 1C and Table 2). We further investigated the differential prognosis between high and low expression levels of PNO1 in three high-risk relapse subgroups: tumor size ≥ 3 cm, high serum AFP level, and more positive rate of Ki-67. Surprisingly, the patients with high PNO1 expression still had the poorest OS and DFS among the three subgroups (Fig. 1H–M).

**Table 2:** Relationship between clinicopathological characteristics and PNO1 expression in 150 HCC patients.

Characteristics		Total	PNO1 expression		*P*-value	Characteristics		Total	PNO1 expression		*P*-value
		150	Low	High				150	Low	High	
Age (years)					0.611	Mavi					0.116
	≥55	74	43	31			Present	16	6	10	
	<55	76	41	35			Absent	134	78	56	
Sex					0.975	Cirrhosis					0.204
	Male	118	66	52			Present	86	52	34	
	Female	32	18	14			Absent	64	32	32	
HBV					0.248	AFP (ng/ml)					0.006*
	Present	116	62	54			≥7	93	44	49	
	Absent	34	22	12			<7	57	40	17	
Tumor size (cm)					0.042*	Staining score of KI67					0.013*
	≥3	98	49	49			≥5%	83	39	44	
	<3	52	35	17			<5%	67	45	22	
Mivi					0.656	BCLC stage					0.191
	Present	81	44	37			0&A	127	74	53	
	Absent	69	40	29			B&C	23	10	13	

Bold and asterisks are used to emphasize that the value is statistically significant.

To verify these findings, we analyzed the bioinformatics database (TCGA and GSE54236). Results revealed that PNO1 expression was higher in tumor tissue than in adjacent normal tissue (Supplementary Fig. 1D and G). Similarly, high PNO1 expression was associated with poorer prognosis of HCC patients based on TCGA (Supplementary Fig. 1E and F). GSEA analysis also showed that high PNO1 expression was positively associated with poor survival in HCC patients (Supplementary Fig. 1H and I). Taken together, these results suggested that PNO1 may be a specific oncogene in HCC.

### PNO1 promoted HCC cell proliferation and inhibited cell apoptosis

**Fig. 2:**
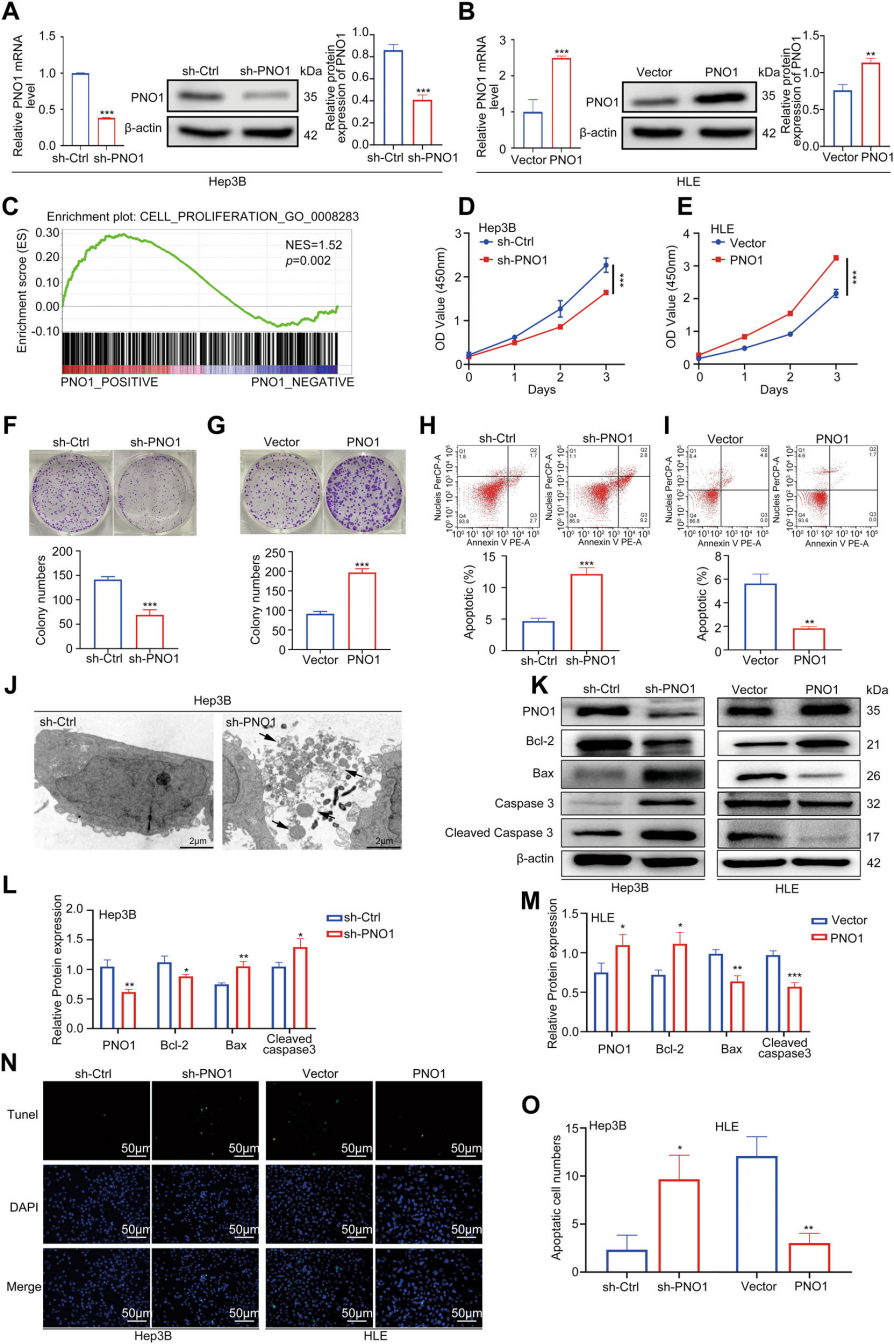
PNO1 promotes HCC cell proliferation and inhibits cell apoptosis. **A**, **B** The construction of Hep3B PNO1- downregulation (sh-PNO1) and HLE PNO1-upregulation (PNO1) cell. **C** GSEA validating the correlation between PNO1 expression and the cell proliferation genes. **D**, **E** CCK-8 assay analysis of the impact of PNO1 knockdown or overexpression on Hep3B and HLE cell growth. **F**, **G** Colony formation assay showing the effects of PNO1 knockdown (**F**) or overexpression (**G**) on Hep3B and HLE cell growth. **H**, **I** Apoptotic rate was measured using annexin V/PI double staining in PNO1 knockdown (**H**) or overexpression (**I**) cells. **J** TEM analysis of cell ultrastructural characteristics in Hep3B sh-PNO1 and sh-Ctrl cells. Chromatin condensation and nuclear fragmentation are indicated by arrows. Scale bar = 20 μm. **K** Western blotting analysis of apoptosis-related protein levels in cells as in (**A**) and (**B**). **L**, **M** Quantification of relative protein expression. **N**, **O** Apoptosis was also evaluated by TUNEL staining (**N**) and quantification of TUNEL positive cells (**O**). Scale bar: 50 μm. Data were presented as mean ± SEM. *n* = 3–4. *p < 0.05, **p < 0.01, ***p < 0.001.

We initially explored the expression level of PNO1 in HCC cell lines and found that PNO1 expression was higher in Hep3B cells and lower in HLE cells (Supplementary Fig. 2A–C). On this basis, Hep3B and HLE cells were used to establish stable PNO1 downregulation (sh-PNO1) and PNO1 upregulation (PNO1) cell lines, respectively. At the same time, we constructed the control cells sh-Ctrl and Vector, respectively. The efficiency of PNO1 deletion and overexpression was confirmed by WB and PCR (Fig. 2A and B). GSEA analysis based on GSE45436 was performed to investigate the biological function of PNO1. The findings suggested that PNO1 expression was positively associated with HCC cell proliferation (Fig. 2C). Then, we assessed the effect of PNO1 on HCC cell viability by using CCK-8, colony formation, and cell apoptosis assays. Results showed that cell viability decreased in Hep3B sh-PNO1 cells (Fig. 2D, F, and H) but increased in HLE PNO1 cells compared with control groups (Fig. 2E, G, and I), respectively. The numbers of apoptosis cells were then observed in Hep3B sh-PNO1 group by transmission electronic microscopy (TEM). The obvious ultrastructure (Fig. 2J) showed more apoptosis bodies in the sh-PNO1 group. To further confirm the effect of PNO1 expression on apoptosis, we detected the expression of apoptosis-related proteins such as Bcl-2, Bax, and activated caspase 3 through Western blotting. The decreased PNO1 expression resulted in decreased Bcl-2 expression but led to increased Bax and activated caspase 3 expression in Hep3B cells. Conversely, PNO1 overexpression showed the opposite effects on these proteins in HLE cells (Fig. 2K–M). Green-stained apoptotic cells were more directly observed in the Hep3B sh-PNO1 and HLE vector groups by TUNEL staining experiment (Fig. 2N and O). Collectively, these findings indicated that PNO1 was an oncogene that promoted HCC cell proliferation and inhibited cell apoptosis.

### PNO1 inhibited cell apoptosis by promoting autophagy in HCC cell line

**Fig. 3:**
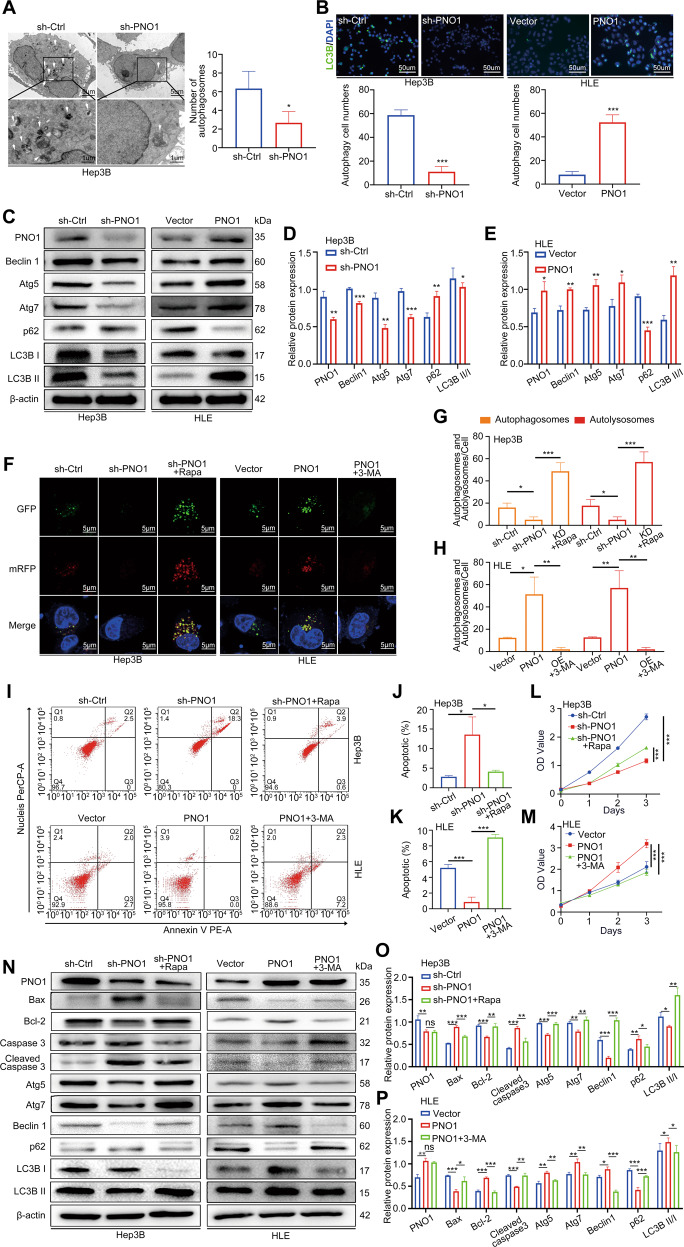
PNO1 inhibits cell apoptosis by promoting autophagy. **A** Autophagy was evaluated using TEM in Hep3B sh-Ctrl and sh-PNO1 cells. The arrows indicate autophagosomes or autolysosomes. Scale bar = 5 μm (upper) or Scale bar = 1 μm (lower). **B** Hep3B sh-Ctrl, Hep3B sh-PNO1, HLE Vector, and HLE PNO1 cells were immunostained with antibodies against LC3B. Scale bar: 50 μm. **C** Western blotting analysis of autophagy-related protein level. **D**, **E** Quantification of relative protein expression. **F**–**H** Hep3B sh-Ctrl, Hep3B sh-PNO1, HLE Vector, and HLE PNO1 cells transfected with GFP-mRFP-LC3 lentivirus. Hep3B sh-PNO1 and HLE PNO1 cells were pretreated with rapamycin and 3-MA, respectively. Images were then acquired by fluorescence microscopy (**F**) and the average number of yellow dots (autophagosomes) or red-only dots (autolysosomes) in the merged images per cell was quantified (**G**, **H**). Scale bar: 5 μm. **I**–**K** Apoptotic rate was measured using annexin V/PI double staining in cells as in (**F**). **L**, **M** CCK-8 assay assessed the viability of cells as in (**F**). **N** Western blotting analysis of apoptosis and autophagy-related protein levels in cells as in (**F**). **O**, **P** Quantification of relative protein expression. Data were presented as mean ± SEM. *n* = 3–4. *p < 0.05, **p < 0.01, ***p < 0.001.

Our TEM data revealed that autophagy levels also decreased in Hep3B sh-PNO1 cells compared with control cells (Fig. 3A). GSEA analysis based on GSE45436 was performed that PNO1 expression was positively associated with autophagy (Supplementary Fig. 2D and E). Then, we assessed autophagosomes by GFP-LC3B dot formation, and the results are shown in Fig. 3B. The number of GFP-LC3B dots in Hep3B sh-PNO1 cells was fewer than those in sh-Ctrl cells. Moreover, more GFP-LC3B dots were present in the HLE PNO1 group than in the vector group. We also detected the expression of autophagy-related proteins via Western blotting. The decreased PNO1 expression resulted in decelerated levels of LC3B II, Beclin1, p62, Atg5, and Atg7 in Hep3B cells, whereas PNO1 overexpression showed the opposite effects on these proteins in HLE cells (Fig. 3C–E). To better understand the effect of PNO1 on autophagy, we utilized the mRFP-GFP-LC3B double-fluorescence system. Results revealed that the numbers of yellow dots (autophagosomes) and red-only dots (autolysosomes) decreased in PNO1 knockdown Hep3B cells, but the numbers of both yellow dots and red-only dots increased upon treatment with the autophagy activator rapamycin (Fig. 3F and G). By contrast, more yellow dots and red-only dots were observed in PNO1-overexpression HLE cells, and this trend was reversed by the autophagy inhibitor 3-MA (Fig. 3F and H). These findings indicated that PNO1 facilitated autophagy flux in cells.

Several studies have shown that autophagy may occur upstream of apoptosis^29,30^. To determine whether PNO1-mediated autophagy is related to apoptosis in HCC cells, we used rapamycin and 3-MA to activate and inhibit autophagy, respectively. When we treated PNO1 knockdown cells with rapamycin to activate autophagy, the apoptosis rate was reduced and cell proliferation was promoted (Fig. 3I, J, and L). Meanwhile, when PNO1-overexpressed cells were treated with 3-MA to inhibit autophagy, the apoptosis rate increased and cell viability was suppressed (Fig. 3I, K, and M). Western blot analysis further showed the same result as depicted in Fig. 3N–P. Taken together, these findings indicated that PNO1 inhibited apoptosis by promoting autophagy.

### PNO1 inhibited cell apoptosis by promoting autophagy through the MAPK signaling pathway in HCC

**Fig. 4:**
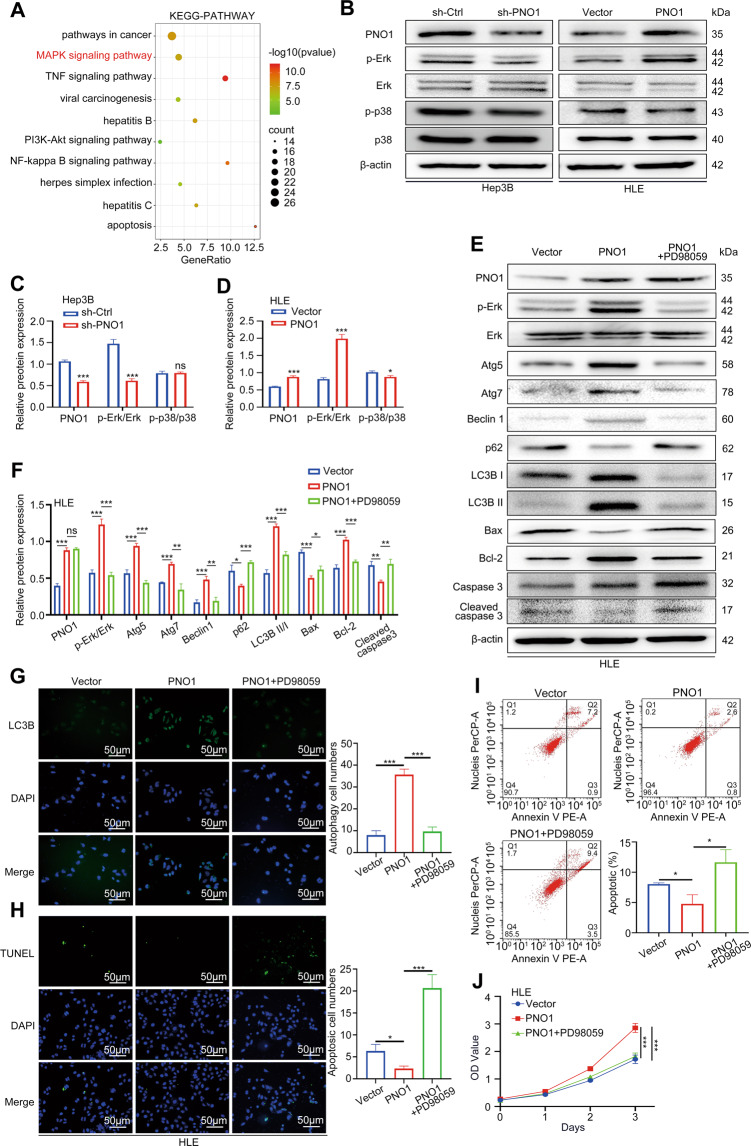
PNO1 inhibits cell apoptosis by promoting autophagy through the MAPK signaling pathway in HCC. **A** Signal pathway enrichment analysis of sh-Ctrl and sh-PNO1 groups by RNA-seq in Hep3B cells. **B** The effect of the downregulation or upregulation of PNO1 expression on MAPK signaling pathway activation was verified by Western blotting in Hep3B and HLE cells. **C**, **D** Quantification of relative protein expression. **E** HLE PNO1 cells were pretreated with PD98059. Western blotting analysis of the protein levels of p-Erk, Erk, and apoptosis and autophagy-related protein. **F** Quantification of relative protein expression. **G**, **H** Cells as in (**E**) were immunostained with antibodies against LC3B (**G**) and TUNEL staining (**H**). Scale bar: 50 μm. **I** Apoptotic rate was measured using annexin V/PI double staining in cells as in (**E**). **J** CCK-8 assay assessed the viability of cells as in (**C**). Data were presented as mean ± SEM. *n* = 3–4. *p < 0.05, **p < 0.01, ***p < 0.001.

To further explore the downstream molecular mechanism of PNO1, we conducted RNA-seq analysis of Hep3B cells with stably downregulated PNO1 expression. With the threshold of a *p*-value < 0.05 and a | log2 FC | > 0.0, a total of 8060 DEGs were detected, including 3914 upregulated DEGs and 4146 downregulated DEGs. GO analysis suggested that PNO1 affected the proliferation and apoptosis of liver cancer cells (Supplementary Fig. 2F). Signal pathway enrichment was then performed based on these DEGs. Notably, in addition to those in pathways involved in cancer, genes in the MAPK signaling pathway were significantly enriched (Fig. 4A). Western blotting revealed that downregulated PNO1 expression suppressed the protein level of p-Erk in Hep3B cells. By contrast, the protein level of p-Erk increased in HLE PNO1 cells (Fig. 4B–D). However, the protein level of p-P38 did not significantly change. To examine the relationship of the MAPK signaling pathway with apoptosis and autophagy, we treated PNO1-overexpression cells with PD98059, which was MEK/Erk inhibitor. p-Erk expression significantly increased in PNO1-overexpression cells but effectively suppressed upon treatment with PD98059 (Fig. 4E and F). Western blotting further showed that the PD98059-mediated inhibition of p-Erk noticeably attenuated the levels of the autophagy-related proteins Atg5, Atg7, Beclin1, p62, LC3B I, and LC3B II and increased the levels of the apoptosis-related proteins Bax, Bcl-2, and cleaved caspase-3 in PNO1-overexpression cells (Fig. 4E and F). We further evaluated autophagy by LC3 immunofluorescence and apoptosis by TUNEL immunofluorescence, Annexin V/PI, and CCK-8 assays. Remarkably, PD98059 treatment significantly inhibited PNO1-induced autophagy (Fig. 4G) and promoted cell apoptosis (Fig. 4H–J). All these results indicated that PNO1 regulated HCC cell’s autophagy and apoptosis through the MAPK/Erk signaling pathway.

### PNO1 promoted LIHC cell tumorigenesis in vivo

**Fig. 5:**
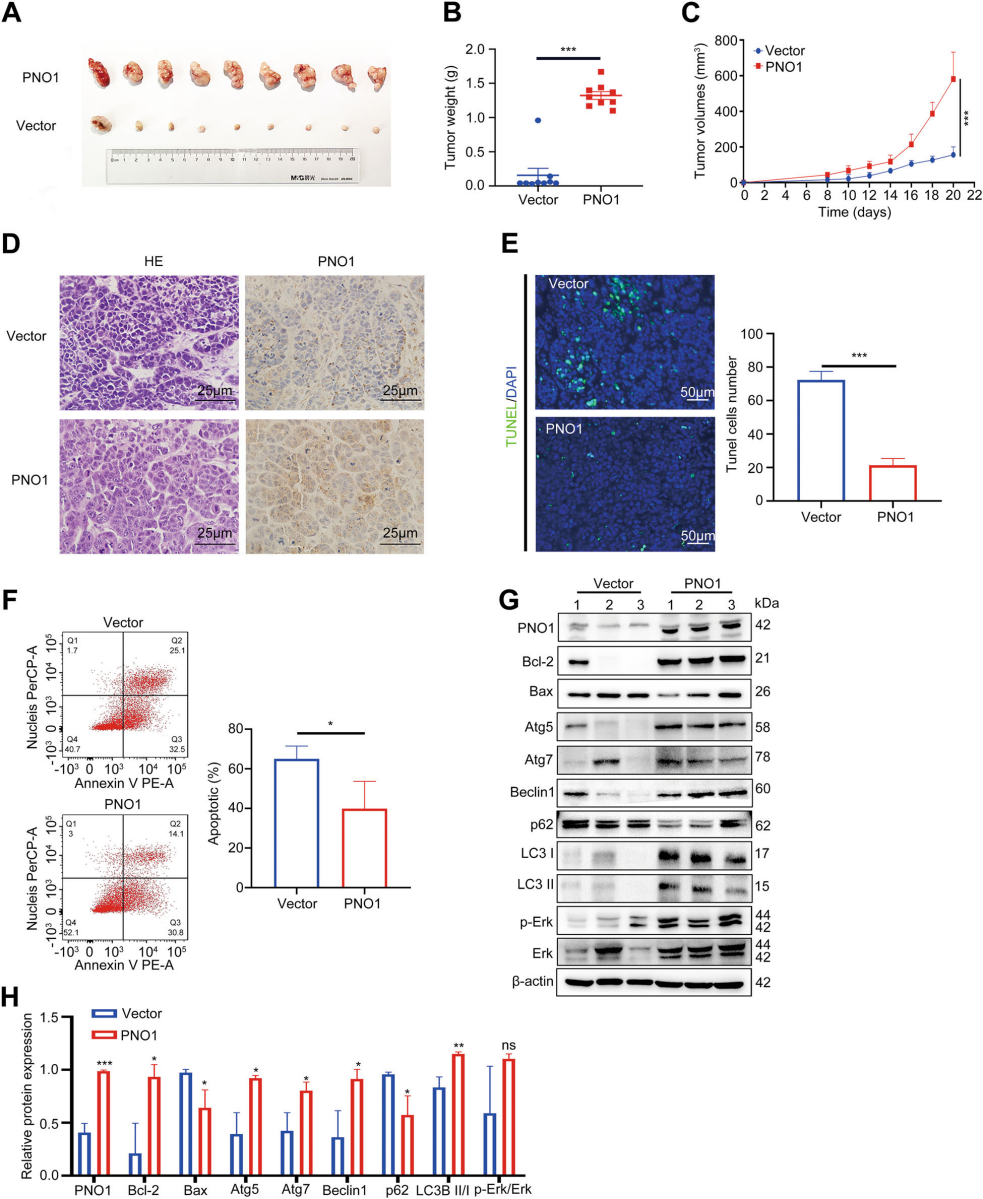
PNO1 promotes HCC tumorigenesis in vivo. **A** Images of tumors from nude mice in HLE Vector and PNO1 groups. **B**, **C** Tumor weights and volumes in the two groups. **D** IHC analysis of PNO1 expression in two groups. Scale bar: 25 μm. **E** Apoptosis was evaluated by TUNEL staining and quantification of TUNEL positive cells in two groups. Scale bar: 50 μm. **F** Apoptotic rate was measured using annexin V/PI double staining in two groups. **G** The protein level of PNO1 and MAPK signaling pathway markers in established xenograft model assessed by Western Blotting. **H** Quantification of relative protein expression. Data were presented as mean ± SEM. *n* = 3–4. *p < 0.05, **p < 0.01, ***p < 0.001.

To further validate the oncogenic activity of PNO1 in vivo, nude mice were subcutaneously injected with HLE Vector cells and HLE PNO1 cells (5 × 10^6^ cells/mouse). Tumor volume was measured every other day from the second week after the injection. All mice were sacrificed at the end of the fourth week, and the primary tumors are shown in Fig. 5A. Tumor weights and sizes were significantly higher in the PNO1 group than in the control group (Fig. 5B and C). Furthermore, we confirmed PNO1 expression in the two groups by immunohistochemistry (Fig. 5D). As shown in Fig. 5E and F, the PNO1 overexpression group had a significantly lower apoptosis rate than the control group. We subsequently examined the indicators related to autophagy and apoptosis through Western blotting (Fig. 5G and H). According to the results of Fig. 4, we considered that PNO1 overexpression activated the MAPK signaling pathway. Thus, we further detected p-Erk and p-P38 expression in the control and high PNO1 expression groups (Fig. 5G and H). Results were consistent with the in vitro ones. Additionally, we observed the same results of smaller tumors and higher apoptosis rates in the Hep3B sh-Ctrl group (Supplementary Fig. 3A–D). In summary, PNO1 overexpression clearly promoted HCC proliferation and autophagy and inhibited apoptosis in vivo.

## Discussion

The development of tumors is always related to the dysregulation of cell proliferation and programmed cell death^31–33^. Apoptosis and autophagy are two kinds of programmed cell death and play crucial roles in tumor progression^34–37^. Accumulating studies have shown that cancer cells usually gain apoptosis resistance resulting in cancer-cell survival and hyperproliferation^38,39^. Activated apoptosis represents a potent cancer-treatment strategy^31–33^. At the same time, imbalance in autophagy plays an important role in cancer cell survival and death and is thus gaining increased attention in cancer therapy^40–42^. In the current work, PNO1 expression was found to be associated with HCC cell apoptosis and autophagy through the MAPK/Erk signaling pathway.

PNO1 as a nuclear protein is involved in ribosome assembly. PNO1 is reportedly responsible for the cleavage of 18S mediated by interaction with Nob1, and both proteins form complexes with the 19S^16,43^. Few studies have been conducted about PNO1 as a potential oncogene that promotes cancer progression. Shen’s group demonstrated that PNO1 may play a critical oncogenic role for human CRC cell-ribosome biogenesis^26,27^. Lin’s group found that PNO1 promotes cell proliferation and inhibits apoptosis in urinary bladder cancer^28^. Pan’s study suggested that PNO1 could be used as a therapeutic target for celecoxib to inhibit HCC and induce tumor proliferation and metastasis^44^. The current study revealed for the first time that PNO1 was overexpressed in HCC tissues and that the molecular mechanism of PNO1 affected the progression of HCC. High PNO1 expression was an independent risk factor in the poor HCC prognosis patients and significantly related to clinicopathological features, such as tumor size, AFP level, and positive rate of Ki-67. In vivo and in vitro experiments revealed that PNO1 strongly promoted autophagy and inhibited apoptosis.

The imbalance between autophagy and apoptosis plays a fundamental role in tissue and organism homeostasis^45,46^. Apoptosis acts as a barrier to inhibit the growth and metastasis of cancer cells^47^. Restoring the apoptotic activity of cancer cells and targeting the anti-apoptotic activity of tumor cells are the current anti-tumor strategies^45,48,49^. The present study showed that HCC cell viability decreased in sh-PNO1 cell lines and apoptosis rate increased. When we used TEM to observe the increase in apoptotic cells in sh-PNO1 cells, we found that autophagosomes dramatically decreased in these cells compared with control cells. Experimental results demonstrated that PNO1 downregulation could increase Caspase-3 activities and the ratio of Bax/Bcl-2. Our results suggested that the retardation of HCC cell proliferation caused by PNO1 downregulation, at least partly, was due to apoptosis induction.

Besides apoptosis, autophagy is also a distinct mode of cell death. Autophagy is associated with the generation of energy and metabolites through the digestion of intracellular macromolecules and organelles. Several studies have indicated that autophagy plays a dual role in cancer^12^. Autophagy, as a suppressor in the early stage of cancer development, inhibits inflammation and promotes genomic stability. Meanwhile, autophagy can promote tumorigenesis and angiogenesis by supplying nutrients and energy^13,14,50,51^. Targeting autophagy as a therapeutic approach for cancer treatment has undergone clinical trials^52,53^. In our research, we showed that PNO1 downregulation interfered with the downstream of autophagy-related markers Beclin-1, p62, Atg5, Atg7, and LC3B. The relationship between autophagy and apoptosis is known to be complex. Accumulating evidence shows that autophagy and apoptosis usually occur within the same cell, and autophagy may serve as upstream regulation mechanism of apoptosis^29,30,54^. According to the different interactions of autophagy and apoptosis in tumor, it could be divided into synergistic, promoting, and antagonistic effects^37,55^. In our experiment, we applied a series of rescue experiments to clarify this point. When we stimulated downregulated PNO1 cells with rapamycin (an autophagy activator), PNO1-knockdown-induced HCC cell proliferation and apoptosis were rescued. The result was reversed after the PNO1 overexpression group was treated with 3-MA (an autophagy inhibitor), leading to increased apoptosis when autophagy was suppressed. Accordingly, we inferred that autophagy contributed to PNO1-mediated HCC cell proliferation and may function upstream of apoptosis.

To investigate the mechanism underlying which PNO1 regulated the apoptosis and autophagy of HCC cells, we performed RNA-seq analysis in shPNO1 and control cells. Results showed that genes in the MAPK signaling pathway were significantly enriched. Several researchers have shown that the activation of the Erk/MAPK signaling pathway could promote the proliferation, invasion, and metastasis of liver cancer cells^56–58^. Furthermore, Erk/MAPK signaling pathway may be a vital pathway of autophagy in cancer^59–61^. In the present study, we found that p-Erk expression changed with PNO1 expression. The inhibitors of the MAPK signaling pathway could dramatically inhibit autophagy and increase apoptosis in PNO1-overexpression cells. Collectively, PNO1 may induce autophagy and inhibit apoptosis through the MAKP signaling pathway in HCC cells.

## Conclusions

In summary, we confirmed that high PNO1 expression was related to poor HCC prognosis by using our clinical data and the TCGA and GEO databases. PNO1 may serve as a specific prognostic biomarker in HCC patients. Our study demonstrated for the first time that PNO1 promoted proliferation and autophagy while inhibiting apoptosis in vivo and in vitro. Furthermore, we found that the MAPK signaling pathway was activated by PNO1 upregulation. All these data highlighted the biological functions of PNO1 and the novel PNO1-mediated mechanism that may be a new target for HCC therapy.

## Supplementary information

supplemental figure-1

supplemental figure-2

supplemental figure-3

## Data Availability

All data are fully available without restrictions.
