# Supplementary figures and images for: PNO1 regulates autophagy and apoptosis of hepatocellular carcinoma via the MAPK signaling pathway
Source: Cell Death Dis. 2021 May 28;12(6):552. doi: 10.1038/s41419-021-03837-y (PMC8163843; doi:10.1038/s41419-021-03837-y)

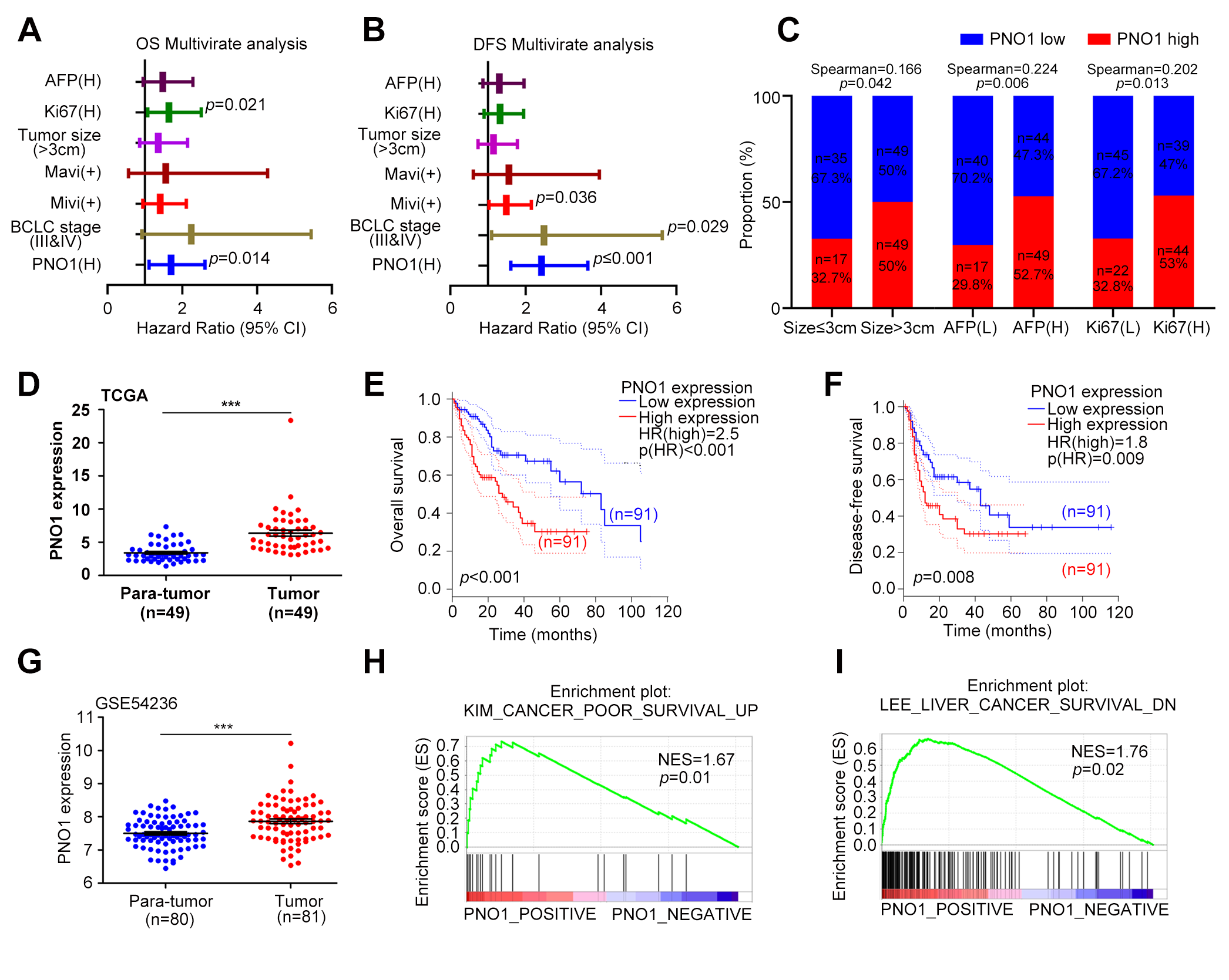

Supplement: Supplementary file 1 — supplemental figure-1 [file 41419_2021_3837_MOESM1_ESM.tif]

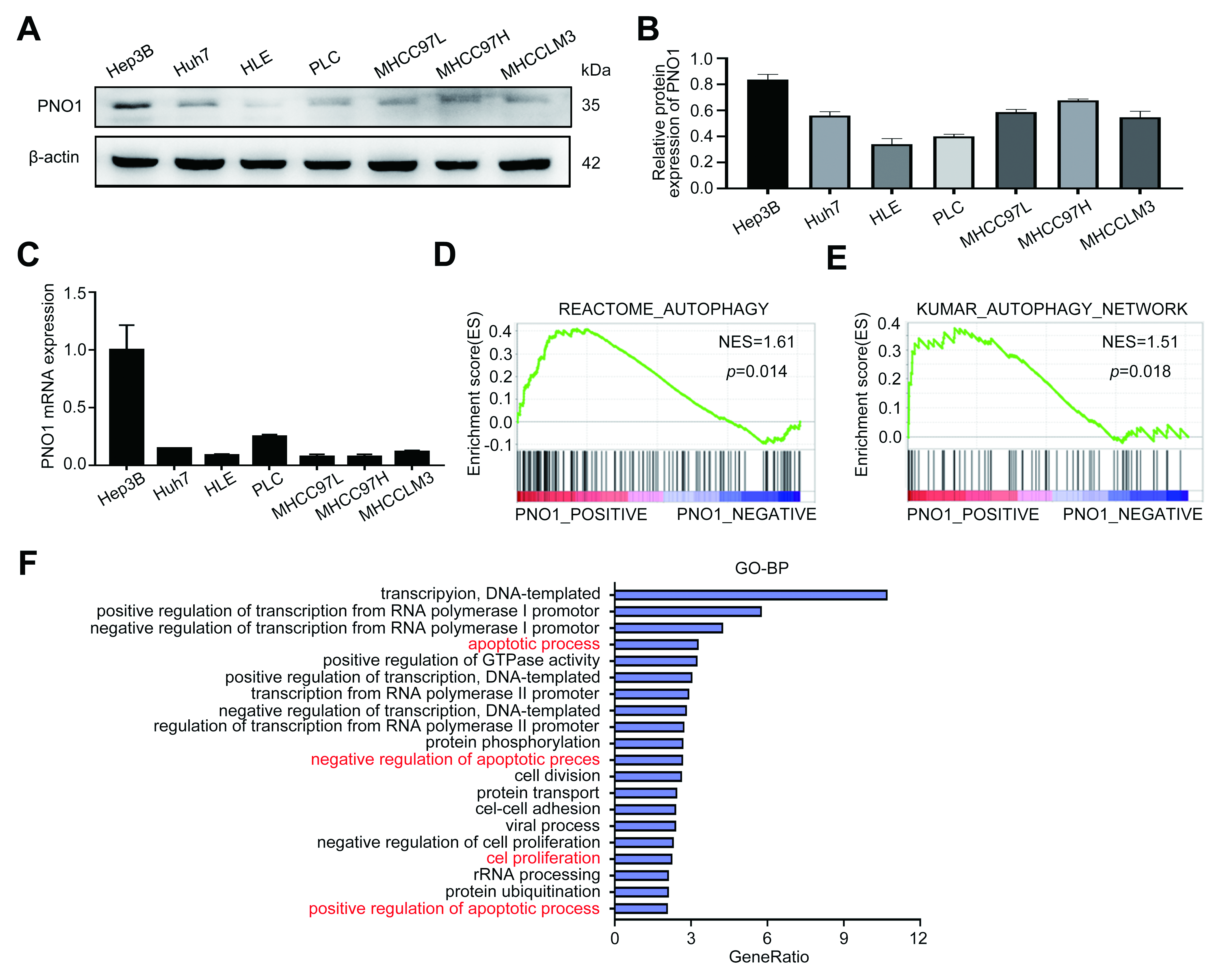

Supplement: Supplementary file 2 — supplemental figure-2 [file 41419_2021_3837_MOESM2_ESM.tif]

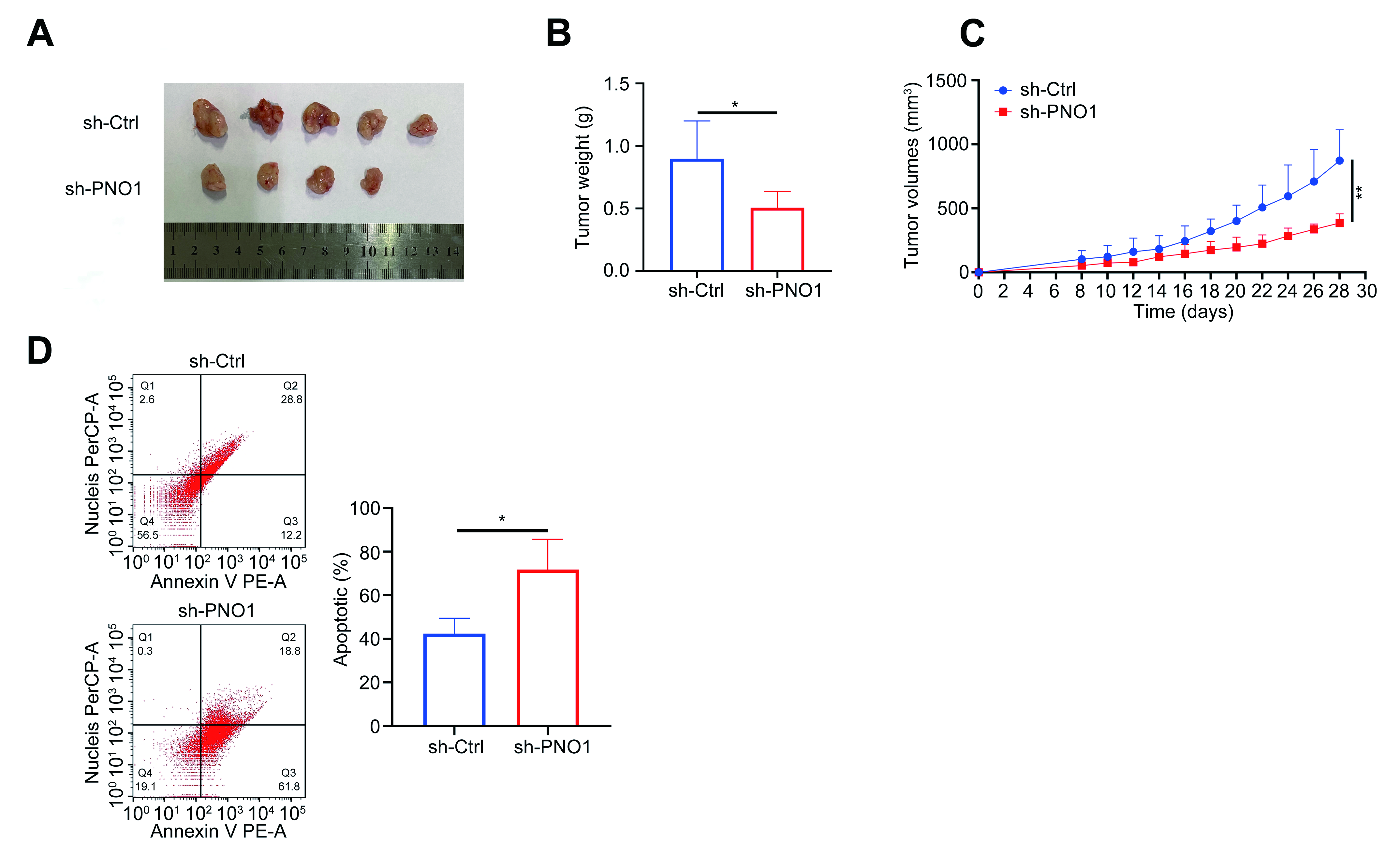

Supplement: Supplementary file 3 — supplemental figure-3 [file 41419_2021_3837_MOESM3_ESM.tif]
